# Influence of microwave processing on nutritional, anti-nutritional, antioxidant and sensory characteristics of kachnar powder and supplemented flatbreads

**DOI:** 10.1016/j.fochx.2024.101888

**Published:** 2024-10-09

**Authors:** Syed Hammad Mazhar, Muhammad Waseem, Zulfiqar Ahmad, Muhammad Rizwan Javed, Muhammad Faisal Manzoor, Muhammad Ammar Khan, Robert Mugabi, Tawfiq Alsulami, Gulzar Ahmad Nayik

**Affiliations:** a Department of Food Science and Technology, Faculty of Agriculture and Environment, The Islamia University of Bahawalpur, 63100, Pakistan; b Guangdong Provincial Key Laboratory of Intelligent Food Manufacturing, School of Food Science and Engineering, Foshan University, Foshan, China; c School of Food Science and Engineering, South China University of Technology, Guangzhou, China; d Department of Food Technology and Nutrition, Makerere University, Kampala, Uganda; e Department of Food Science and Nutrition, College of Food and Agriculture Sciences, King Saud University, P.O Box-800, Riyadh 11451, Saudi Arabia.; f Marwadi University Research Centre, Department of Microbiology, Marwadi University, Rajkot 360003, Gujarat, India.

**Keywords:** *Bauhinia variegata*, Flowers, Thermal processing, Value addition, Fortification, Flatbreads

## Abstract

This study examines the impact of microwave processing on Kachnar (*Bauhinia variegata*) powder and its application in flatbreads. Microwave treatment reduced anti-nutrient levels of alkaloids, phytates, tannins, and saponins by 83–90 %, enhancing safety. Incorporation of microwaved kachnar powder (MwKP) into flatbreads at 2.5–10 % replacement levels improved nutritional profiles, with increases in ash (0.4–0.9 g/100 g), dietary fiber (2–3 g/100 g), protein (8–9 g/100 g), and minerals such as Fe (3–4 mg/100 g), Zn (3.2–3.9 mg/100 g), Na (3–10 mg/100 g), K (378–388 mg/100 g), Ca (30–45 mg/100 g), and Mg (125–145 mg/100 g). Antioxidant activity also increased significantly (*p* < 0.05) as measured by DPPH, ABTS, FRAP, and total phenolic and flavonoid contents. Sensory evaluation showed a decline in acceptability for MwKP levels ≥7.5 %, though 5 % supplementation was well-received. The findings suggest that microwave processing is an effective method for reducing anti-nutrient content in Kachnar while improving its nutritional and antioxidant properties, making it a viable ingredient for enhancing baked goods.

## Highlights

• Microwave processing at 0.8 kW, 3 min reduced antinutrients in *B. variegata* by 90 %.
• Higher Fe, Zn, Ca, fibers & proteins were reported in RKP & supplemented flatbreads.
• RKP elucidated higher concentrations of phenolics, flavonoids & antioxidants.
• Consumers preferred MwKP supplemented flatbreads at 5 % for all sensory attributes.

## 1 Introduction

Kachnar (*Bauhinia variegate* Linn.), also known as mountain ebony, orchid tree, camel's foot is botanically a flowering plant in the family Fabaceae. It is native to Southern China, Southeast Asia, and the Indo-Pakistan region (Kamal et al., 2022). The foliar composition of kachnar reflects its fresh, pleasant fragrance and contains a variety of health promoting phytonutrients including micro and macro-nutrients, dietary proteins and beneficial dietary fibers. It is renowned for its nutrient and energy dense attributes among the flowering vegetables. Additionally, kachnar's edible flowers are recognized as a viable carrier of polyphenols, reducing sugars and inorganic residues, vitamin C, and essential fatty acids such as linoleic, linolenic, oleic, stearic and palmitic acids (Sharma et al., 2021). This nutrient dense vegetable also contains substantial concentrations of health promising bioactive compounds such as kaempferol, carotenoids, flavonoids, hesperidin, flavanol glycosides, triterpene caffeate, ombuin, flavanone, quercetin, anthocyanin, rutin, and germacrene D (Sharma et al., 2021). These compounds have been reported to exhibit several biological activities such as anti-oxidative, anti-cancer, anti-worm, antitumor, anti-ulcer, anti-microbial, anti-diabetic, anti-leukemia, anti-ulcer, anti-hemagglutinating (Ali et al., 2021; Khare et al., 2018), anti-bronchitis, immunomodulatory, anti-dysentery, anti-inflammation, anti-skin disorders, hepatoprotective, anti-diarrhea, anti-hypolipidemic, anti-leprosy, and anti-wound properties (Bhandari et al., 2017; Kansal et al., 2020). These health benefits are linked with the higher concentrations of biologically active nutrients including polyphenols and flavonoid in kachnar (Tripathi et al., 2019).

Antinutrients are plant antimetabolites which emerge as a response by the defense mechanism system in plants to protect plants from any harsh or unfavorable conditions (López-Moreno et al., 2022). On dietary exposure, these toxic compounds adversely affect the human health by altering the biochemical reactions and also cause mineral bioavailability issues (Faizal et al., 2023). Earlier available literature validates, antinutrients not only impair mineral bioavailability but can also lead to serious health challenges such as kidney stones and renal failure (Waseem et al., 2021). The presence of anti-nutritional and gas producing intrinsic toxicants in vegetables pose significant challenge to their edibility. Despite of remarkable nutritional significance, kachnar is also known to contain substantial levels of these toxicants such as alkaloids, tannins, phytates, saponins, trypsin inhibitors, and oxalates (Seed & Balogun, 2013; Thakur et al., 2020). Scientists are actively seeking viable effective processing techniques to mitigate antinutrients in vegetables at domestic and industrial levels. Earlier research has revealed that various processing techniques, including thermal (i.e., autoclaving and boiling) and non-thermal (i.e., germination, fermentation, soaking, and extrusion), can help to reduce the antinutrients loads in vegetables (Waseem et al., 2024). Focusing on the challenges such as nutrient losses, high costs, reduced sustainability, and waste generation associated with the current techniques, researchers are exploring alternative, more efficient, cost-effective, and sustainable methods. Microwave processing, is being explored as a potential processing method to reduce the loads of antinutrient, while enhancing the nutritional value of vegetables. Also, present study preferred the microwave processing over other techniques to process kachnar owing to its higher efficiency to reduce toxic antinutrients, its better nutrient and sensory retention attributes (Suhag et al., 2021; Waseem et al., 2022b). Earlier studies have validated the higher potential of microwave heat processing in decreasing the antinutrients in vegetables on comparison with the other processing techniques such as blanching and soaking and elucidated 85–90 % reductions (Waseem et al., 2022b; Waseem et al., 2024).

In recent decades, there has been significant focus on development of value-added food products from nutrient dense natural ingredients to meet the dietary needs of ever-increasing population. Foliar parts of Kachnar known for their nutrient rich profile, are culturally used as viable ingredients of choice in development of value-added products including curries, pickles, pakoras and chutneys (Sharma et al., 2021), savory and sweet dishes like kachnar biryani and sherbet, kachnar herbal extracts, kachnar juice and thyroid capsules (Kansal et al., 2020). Flatbreads (i.e., chapatis) are among the oldest bakery products consumed worldwide and valued for their unique nutritional attributes. These baked goods are more preferred in South-east Asia, Indian subcontinent, North and South Africa, Middle East, Europe, China and Central America (Waseem et al., 2022b). Consumer dietary preferences for the flatbreads are linked with the ease of preparation, convenience, and nutritional advantage as an affordable staple food. Consequently, the global consumption of flatbreads is transitioning from traditional methods of production to more mechanized and commercial systems.

Therefore, this study aims to mitigate the levels of antinutrients in kachnar powder using microwave processing. It also explores the potential for adding value to kachnar powder in the development of unleavened flatbreads and assesses their nutritional, antioxidant, and organoleptic attributes for maximum acceptability. Additionally, the study highlights the differences in key attributes between flatbreads made with refined wheat flour and those made with MwKP.

## 2 Materials and methods

### 2.1 Raw materials, chemicals and reagents procurement

Fresh Kachnar flowers were randomly harvested from the local kachnar fields available at the Islamia University, Bahawalpur, Punjab, Pakistan. Refined wheat flour was purchased from the local market of Bahawalpur, Punjab, Pakistan. The reagents and chemicals including DPPH (2,2-diphenyl-1-picryl-hydroxyl) reagent, sodium acetate buffer (pH ~ 3.6), Folin-Ciocalteu reagent (FCR), thioglycolic acid, 2,2′-bipyridine, tannic acid, ABTS solution, 2,2-pyridyl, ascorbic acid, gallic acid and TPTZ (2, 4, 6-tri 2-pyridyl-*s*-triazine), nitric acid (HNO_3_), sodium acetate buffer, thioglycolic acid, and hydrochloric acid (HCl) were sourced from Sigma-Aldrich Inc., USA.

### 2.2 Preparation of raw kachnar powder (RKP) and MwKP

After sorting and grading, insect infestation free and fresh flowers' petals were microwave processed at 0.8 kW for 3 min (Waseem et al., 2024). Thereafter, the raw and microwave processed kachnar flower petals were placed in cabinet dryer (Pamico Technologies, Faisalabad, Punjab, Pakistan) at 45 °C for 10 h for drying to a moisture content of about 9–13 %. Following dehydration, the flower petals were ground into fine powder with a mesh size of about 70 mm. Both RKP and MwKP samples were filled in airtight glass jars and stored at room temperature for further appraisal in the study.

### 2.3 Product development

#### 2.3.1 Preparation of refined wheat flour (RWF) and MwKP supplemented flatbreads

Flatbreads were prepared following the method as described by (Hussnain et al., 2022). Flour premix blends were prepared by replacing RWF with MwKP at 2.5–10 %. The premix blends i.e., T_0_ (100 % RWF, control), T_1_ (97.5 % RWF + 2.5 % MwKP), T_2_ (95 % RWF + 5 % MwKP), T_3_ (92.5 % RWF + 7.5 % MwKP), and T_4_ (90 % RWF + 10 % MwKP) were taken in pre-made proportions and kneaded into dough (50 g each) using the laboratory dough maker (HA-3480AS, Shenzhen, China). Following kneading, the dough samples were rested for 30 min and converted into thin sheets with a diameter of 12 cm and thickness of 2 mm using roller pin. The sheets were evenly baked on both sides at 200 ± 5 °C for 1–2 min using a spherical griddle.

#### 2.3.2 Nutritional and mineral composition determination in RWF, RKP, MwKP and MwKP supplemented flatbreads

Moisture (925.10), ash (923.03), fat (920.85), fiber (32−10) and protein (920.87), micro and macro-minerals of RWF, RKP, MwKP, and supplemented flatbreads were determined in accordance with the standardized protocols as mentioned by the Association of Official Analytical Chemists, Latimer Jr (2019). However, carbohydrate contents as nitrogen free extracts (NFE) were estimated using following formula;

(I) $$\mathrm{NFE}=100-\%\left(\text{moisture}+\mathrm{ash}+\text{protein}+\mathrm{fat}+\text{fiber}\right)$$

### 2.4 Antinutrients determination in RWF, RKP, MwKP and MwKP supplemented flatbreads

#### 2.4.1 Phytates contents

Phytates contents in RWF, RKP, MwKP and supplemented flatbreads were measured by following the method as described by (Waseem et al., 2024). Accurately measured 10 mL of 0.2 N hydrochloric acid (HCl) was mixed in one gram of each sample with continual stirring for 30 min. Subsequently, 0.5 mL of the resultant extract was added into 1 mL of ammonium iron sulfate solution, followed by boiling for 30 min and rested for 20 min. The resultant mixture was centrifuged (Hermle Z236K, Wehingen, Germany) at 1107*g* for 30 min. Afterwards, about 1 mL of the supernatant was transferred into a volumetric flask already containing 1.5 mL of 2,2′-bipyridine solution (prepared by mixing 0.25 g each of thioglycolic acid and 2,2′-bipyridine with a total volume of 25 mL). The spectrophotometric absorbances (UV–Vis 3000, Darmstadt, Germany) of the reagent blank and samples were measured at 519 nm using phytate-phosphorous as standard (100–1000 mg/L).

#### 2.4.2 Alkaloid contents

Alkaloids were determined following the method as described by (Waseem et al., 2024). Precisely measured 5 g of each sample was mixed in 50 mL of 10 % (*v/v*) acetic acid solution using ethanol as solvent. The reaction mixture was incubated for 4 h at room temperature. The contents were filtered using Whatman filter paper (No. 41). Alkaloid residues were precipitated by titrating the filtered solution with concentrated ammonium hydroxide (NH_4_OH). Subsequently, a secondary filtration was done followed by second washing of alkaloid precipitates with 1 % ammonium hydroxide solution. The resulting precipitates on the filter paper surface were dried in a hot air oven at 60 ± 2 °C for 30 min. The oven dried alkaloid precipitates along with filter paper were then weighed and measured for alkaloids contents using following formula;

(II) $$\text{Alkaloids}\;\left(\mathrm{mg}/100\;g\right)=\frac{\mathrm{Weight of alkaloid precipitates with filter paper}\;\left(g\right)-\mathrm{Weight of filter paper}\left(g\right)}{\mathrm{Weight of sample}\;\left(g\right)}\times 100\;$$

#### 2.4.3 Tannin contents

RWF, RKP, MwKP and supplemented flatbreads were determined for tannins by flowing the method as previously reported by (Ifeanacho & Ezecheta, 2020). Accurately weighed 250 mg of each sample was mixed in 25 mL of distilled water in 250 mL conical flasks and shaken using the orbital shaker (MaxQ™ 4000, Thermo Scientific, Waltham, MA, USA) for 1 h. The admixture solution was subjected to filtration in a 25 mL volumetric flask and filled up to the mark. From the filtrate, 2.5 mL was mixed in 0.1 mol/L HCl, 0.008 mol/L K_4_Fe(CN)_6_ (potassium ferrocyanide) and 1 mL of 0.1 mol/L ferric chloride (FeCl_3_). Thereafter, within the early 10 min of the mixture solution preparation, spectrophotometric absorbance (UV–Vis 3000, Darmstadt, Germany) of each sample, reagent blank and tannic acid standards (100–1000 mg/L) were measured at 605 nm. Tannins contents were measured using the following formula;

(III) $$\text{Tannin contents}\;\left(\mathrm{mg}/100\;g\right)=\frac{\text{Conc of standard}\;\left(\frac{\mathrm{mg}}{L}\right)\times \mathrm{Abs}\;\text{of Filtrate}\times \text{Total}\;\mathrm{vol}\;\text{of filtrate}\;\left(\mathrm{mL}\right)\times 1000\;}{\mathrm{Abs}\;\text{of standard}\times \text{Weight of sample}}\times 100$$

#### 2.4.4 Saponin contents

Saponin contents in RWF, RKP, MwKP and supplemented flatbreads were determined by using the standard protocol as laid down by (Stoleru et al., 2022). Accurately measured 2.0 g of each of sample was extracted by refluxing in a Soxhlet extractor (2055 Soxtec Foss Tecator, Shandong, China). Earlier, extraction process was separately carried out using 200 mL each of acetone and methanol for 3 h each. After methanol extraction, the sample was dried in the oven and later allowed to cool at room temperatures, before it was weighed. The concentration of saponin was analyzed based on the following formula;

(IV) $$\text{Saponin}\;\left(\mathrm{mg}/100\;g\right)=\frac{\mathrm{Weight of flask with extract}-\mathrm{Weight of empty flask}}{\text{Weigh of sample}}\times 100$$

### 2.5 Antioxidants activities determination in RWF, RKP, MwKP and MwKP supplemented flatbreads

#### 2.5.1 Free radical scavenging activities (DPPH)

A method of (Khan et al., 2023) was followed to determine free radical scavenging activities in RWF, RKP, MwKP, and supplemented flatbreads. About 2 g of each sample was mixed in precisely measured 10 mL ethanol followed by stirring for 40 min at 4 °C After stirring, the admixture was centrifuged (10,000 ×*g*) for 10 min at 4 °C to reserve the supernatant.

Following equation was used to estimate the free radical scavenging activities as;

(V) $$\text{Radical scavenging activities}\;\left(\%\right)=\frac{\text{Absorbance of blank}-\text{Absorbance of sample}}{\text{Absorbance of blank}}\times 100$$

#### 2.5.2 Frap

FRAP of all samples was assessed using the procedure as outlined by (Bratovcic et al., 2021). The FRAP reagent was prepared by mixing 200 mL of sodium acetate buffer solution (300 mmol/L, pH 3.6), 20 mL tripyridyl triazine (TPTZ) solution (10 mmol/L in 40 mmol/L HCl), 20 mL FeCl_3_ solution (20 mmol/L), and 24 mL of distilled water. A mixture of 0.2 mL of methanol extract and 3.8 mL of FRAP reagent was incubated for 4 min at ambient temperatures. Spectrophotometer (UV–Vis 3000, Darmstadt, Germany) absorbances of each sample, reagent blank and standards were recorded using the Spectrophotometer (UV–Vis 3000, Darmstadt, Germany) at 593 nm. The control was consisted of 0.2 mL of distilled water and 3.8 mL FRAP reagent. FRAP of all samples were measured in mmol FeSO_4_ equivalents per 100 g of dry weight sample.

#### 2.5.3 ABTS

For ABTS assay, a solution was prepared by mixing equal parts of 7 mM ABTS and 2.45 mM potassium persulfate, which was then kept in dark for 16 h. The mixture was diluted using ethanol until the absorbance reached 0.70 (± 0.02) at 734 nm. Subsequently, 0.02 mL of the extract was combined with 0.08 mL of distilled water and 3 mL of the ABTS solution, and the mixture was allowed to react for 15 min. The absorbance was measured at 734 nm using a UV–Vis spectrophotometer (UV–Vis 3000, Darmstadt, Germany). The antioxidant activity of the extract was determined and expressed in μmol TE/g as described by (Weremfo et al., 2022).

#### 2.5.4 TPC

Folin-Ciocalteu reagent method was used to estimate the TPC in RWF, RKP, MwKP and MwKP supplemented flatbreads by following the method as described by (Park et al., 2022). Precisely measured 0.3 mL of each sample extract was mixed in 2.5 mL of 10 % Folin-Ciocalteu reagent and was rested for 5 min. Thereafter, 2.5 mL of 6 % sodium carbonate solution was added and now rested for 90 min. Now, the absorbance of each sample and reagent blank was measured at 725 nm using spectrophotometer (UV–Vis 3000, Darmstadt, Germany). Gallic acid standard curves (1–1000 mg/L) were plotted and results were recorded in mg GAE/g.

#### 2.5.5 TFC

The aluminum chloride colorimetric assay was followed to determine the total flavonoid content of each sample by adopting the method of (Wu et al., 2021). A UV–Visible spectrophotometer (UV–Vis 3000, Darmstadt, Germany) was used to measure absorbance at 510 nm for both the sample and reagent blank. TFC mean values were expressed as mg CAE/g against the standard curves of catechin as 10–1000 mg/mL.

### 2.6 Determination of carotenoids, tocopherols texture and chroma in RWF, RKP, MwKP and MwKP supplemented flatbreads

#### 2.6.1 Carotenoids

Carotenoid contents of RWF, RKP, MwKP, and supplemented flatbreads were determined as described by (Thakur et al., 2020). About 10 g of each sample was saponified for approximately 30 min using a shaking water bath (SBS40, Stuart, UK) maintained at 37 °C, then extracted with alcoholic potassium hydroxide. Now the saponified extract was transferred in to a separating funnel which already contained 15 mL petroleum ether and was mixed thoroughly. On mixing, the carotenoid pigments were carried towards petroleum ether layer. Now, the upper petroleum ether layer containing carotenoids were transferred to an amber colored bottle, while the lower aqueous layer was separated using separating funnel. Aqueous layer was re-extracted to obtain a colorless layer indicating the absence of carotenoids in aqueous layer. The aqueous layer was disposed off and sodium sulphate was added in the petroleum ether upper layer to remove any turbidity. The final volume of upper petroleum ether layer was recorded and absorbance of each sample and reagent blank was taken at 450 nm using Spectrophotometer (UV–Vis 3000, Darmstadt, Germany).

(VI) $$\text{Carotenoids}\;\left(\mathrm{\mu g}/g\right)=\frac{\text{Optical density of the sample}\times \text{Volume of sample}\times 4}{\text{Weight of sample}}\times 100$$

#### 2.6.2 Tocopherols

RWF, RKP, MwKP, and MwKP supplemented flatbreads were analyzed for tocopherols by using the method as described by (Thakur et al., 2020). Accurately measured 100 g each sample was mixed with 0.1 N sulfuric acid and allowed to rest for 12 h and filtered. Afterwards, 1.5 mL of tissue extract and 1.5 mL of xylene were mixed and centrifuged. Subsequently, 1 mL of xylene was centrifuged and mixed with 1 mL of 2,2-pyridyl, then the optical density was observed at 460 nm. In the blank solution, 0.3 mL of ferric chloride (FeCl_3_) was added with constant stirring and allowed to settle. Absorbance of test and standard samples were recorded at 520 nm with reference to the blank. Tocopherol contents in all samples and reagent blank were estimated using following formula;

(VII) $$\text{Tocopherol}\;\left(\mathrm{\mu g}/g\right)=\frac{\text{Reading}\;\mathrm{at}\;520\mathrm{nm}–\text{Reading}\;\mathrm{at}\;450\mathrm{nm}}{\;\text{Reading of standard}\;\mathrm{at}\;520\mathrm{nm}\;X\;0.29\;X\;15}$$

### 2.7 Texture and puffing height estimation of RWF and MwKP supplemented flatbreads

The textural attributes i.e., hardness (N), gumminess (N, mm), and springiness (mm) of RWF and MwKP supplemented flatbreads were determined using texture analyzer (TA. XT plus, Stable Micro Systems). Likewise, the puffing heights of RWF and MwKP supplemented flatbreads were measured in cm using clean stainless-steel scale by following the method as outlined by (Waseem et al., 2021).

### 2.8 Sensory evaluation of RWF and MwKP supplemented flatbreads

Sensory analysis consisting of texture, color, taste, appearance and folding ability of RWF and MwKP supplemented flatbreads was performed by semi-trained but experienced (i.e., minimum five years) panel consisting of 20 faculty members both male & female holding PhD degrees in Food Science and Technology and PhD scholars (both male & female) at the Department of Food Science & Technology, Islamia University of Bahawalpur, Punjab, Pakistan. 9-point hedonic scale ranging between 1 and 9 from extremely disliked to extremely liked (Kiumarsi et al., 2019). The sensory analysis was performed without any biasness. The panelists were also provided with the sufficient amount of clean drinking water, ample light and odor free environment. Further, the panelists were also briefed about the work done in this study and they were told how the end product was going to be evaluated. All sensory panelists were taken on board for their consent to participate in this study.

### 2.9 Statistical analysis

All analyses were carried out in duplicates, and the results were documented as mean ± standard deviation (S.D.). The nutritional, antinutrients, antioxidants and textural quality parameters of RWF, RKP, MwKP and supplemented flatbreads were analyzed using the Analysis of Variance (ANOVA) on Statistix 8.1 (Tallahassee, FL, USA). The least significant difference (LSD) test was used employed in testing the level of significance among the means for all parameters at 0.05 confidence interval.

## 3 Results and discussion

### 3.1 Nutritional composition of RWF, RKP, MwKP and MwKP supplemented flatbreads

Proximate composition of raw and processed kachnar powders depicted the highest ash, fiber and protein contents in MwKP i.e., 4.6, 9.8 and 9.5 g/100 g, respectively (Table 1). Higher ash and fibers in microwave processed samples might be due to thermal degradation and decrease in moisture on microwave heating of kachnar (Sharma et al., 2022). Similarly, another study by (Sharma et al., 2021) elucidated matchable contents for ash, fiber and protein in kachnar powder i.e., 5, 8.6 and 2.5 g/100 g, respectively. The results for MwKP supplemented flatbreads showed significant (*p* < 0.05) enhancement in ash, fiber, and protein contents from 0.4 to 0.9, 2–3 and 8–9 g/100 g, respectively at 0–10 % (T_0_-T_4_) supplementation levels. However, significant increase in proximal contents of MwKP supplemented flatbreads could be attributed to the presence of higher magnitudes of these constituents in RKP itself (Table 1). The results for the moisture and fat contents elucidated notable decline from 26 to 24 and 1.6–1.4 g/100 g, respectively on supplementing the MwKP in flatbreads at 0–10 %. Our results for the proximal contents of baked good are slightly different with an earlier study by (Gupta et al., 2017) wherein the researchers elucidated higher contents of ash and proteins i.e., 21.4 and 4.3 g/100 g, respectively at 10 % kachnar leaf powder supplementation levels in *Chilas* (i.e., Indian indigenous baked product). The change in proximal contents could be linked with the variety in cultivar, species and environmental conditions. Likewise, another study of researchers consisted on (El-Sohaimy et al., 2019) also reported the presence of higher magnitudes of ash, fiber and proteins i.e., 1.6–2.8, 0.5–1.8, and 13.7–15.8 g/100 g, respectively in quinoa-wheat flour composite flatbreads. Hence, kachnar powder, being a potential carrier of dietary fibers can provide value added baked products with better fibers and proteins.

**Table 1:** Nutritional composition of RWF, RKP and MwKP and MwKP supplemented flatbreads (g/100 g).

**Powders**	**Moisture**	**Ash**	**Fat**	**Fiber**	**Protein**	**NFE**^**⁎**^
RWF	11.26 ± 0.10^b^	0.47 ± 0.03^c^	1.39 ± 0.07^b^	2.54 ± 0.10^c^	8.28 ± 0.07^b^	76.06 ± 0.23^a^
RKP	13.17 ± 0.86^a^	3.36 ± 0.16^b^	2.18 ± 0.07^a^	7.69 ± 0.19^b^	7.17 ± 0.04^c^	66.45 ± 0.94^b^
MwKP	9.84 ± 0.08^b^	4.60 ± 0.07^a^	1.22 ± 0.02^b^	9.83 ± 0.07^a^	9.51 ± 0.06^a^	65.01 ± 0.10^b^

**Flatbreads**	**Moisture**	**Ash**	**Fat**	**Fiber**	**Protein**	**NFE**^**⁎**^
T₀	26.44 ± 0.01^a^	0.44 ± 0.01^e^	1.65 ± 0.07^a^	1.95 ± 0.07^e^	8.05 ± 0.35^d^	61.47 ± 0.24^a^
T₁	26.01 ± 0.01^b^	0.56 ± 0.02^d^	1.58 ± 0.02^ab^	2.23 ± 0.05^d^	8.29 ± 0.01^cd^	61.33 ± 0.11^ab^
T₂	25.64 ± 0.05^c^	0.68 ± 0.02^c^	1.54 ± 0.01^b^	2.46 ± 0.03^c^	8.56 ± 0.05^bc^	61.13 ± 0.04^ab^
T₃	24.78 ± 0.15^d^	0.80 ± 0.03^b^	1.52 ± 0.02^b^	2.72 ± 0.05^b^	8.79 ± 0.04^ab^	61.40 ± 0.04^ab^
T₄	23.94 ± 0.08^e^	0.91 ± 0.0 + 2^a^	1.42 ± 0.02^c^	3.02 ± 0.12^a^	9.10 ± 0.14^a^	61.62 ± 0.18^a^

Values are expressed as means ± S.D. (*n* = 2). Mean values presenting similar lettering in a column are statistically non-significant at *p* < 0.05. T_0_ = 100 % RWF flatbreads (Control), T_1_ = 2.5 % MwKP, T_2_ = 5 % MwKP, T_3_ = 7.5 % MwKP, T_4_ = 10 % MwKP flatbreads. ^⁎^Nitrogen-free extract = 100 – g/100 g (moisture + ash + fat + fiber + protein).

### 3.2 Mineral composition of RWF, RKP, MwKP and MwKP supplemented flatbreads

The results for the mineral composition of RKP and MwKP elucidated the highest concentrations of Fe, Zn, Na, K and Ca in MwKP i.e., 8.4, 6.8, 62, 90 and 144 mg/100 g, respectively when compared with the RKP and control (Table 2). However, the elevated magnitudes of these inorganic residues in the microwave heat treated samples could be attributed to a number of interacting mechanisms including thermal degradation, weaker bonding and reduced moisture contents of the powders itself as earlier suggested by (Bhavya & Prakash, 2021). The results for the mineral profile of kachnar in present investigation are in close harmony with the earlier researchers consisting of (Awasthi & Verma, 2019) wherein, the researchers reported the presence of Fe, Zn, Na, K and Ca in kachnar powder as; 10, 5, 55, 83 and 134 mg/100 g, respectively. The results for MwKP supplemented flatbreads revealed significant (*p* < 0.05) increase in micro and macro elements i.e., Fe, Zn, Na, K, and Ca from 3.1 to 4, 3.2–3.9, 2.9–9.6, 378–388, and 30.2–45.3 mg/100 g, respectively (Table 2). A successive increment in all the minerals was observed in flatbreads on gradual increase of the supplementation levels of MwKP in the baked goods from 0 to 10 % (i.e., T_0_-T_4_). However, among the treatment groups T_4_ showed the highest contents of Fe, Zn, Na, K, and Ca at maximum MwKP supplementation level (i.e., 10 %), when compared with the control (i.e., T_0_) which reported the lowest values of these minerals. Comparable findings for the mineral contents were reported by (Gupta et al., 2017) wherein the researchers elucidated significant enhancement in Fe and Ca concentrations of *Chila* from 2.1 to 2.2 and 45–47 mg/100 g, respectively on replacement of wheat flour with kachnar leaf powder at 10 % supplementation levels. Similarly, in another recent study the scientific explorers reported a measurable increase in Fe contents of functional biscuits on replacing the wheat flour with kachnar leaf powder (Ahmed et al., 2023). However, another research by (Elfeky et al., 2024) found noticeable increment in Zn and Ca levels in sheeted pancakes from 2.8 to 4.9 and 259–268 mg/100 g, respectively on substitution of 10 % lupin flour with sorghum flour.

**Table 2:** Mineral composition of RWF, RKP, MwKP and MwKP supplemented flatbreads (mg/100 g).

**Powders**	**Fe**	**Zn**	**Na**	**K**	**Ca**	**Mg**
RWF	3.66 ± 0.13^c^	3.88 ± 0.04^c^	5.25 ± 0.10^c^	438.00 ± 5.19^a^	39.76 ± 0.85^c^	131.00 ± 1.41^c^
RKP	7.88 ± 0.03^b^	5.64 ± 0.11^b^	58.29 ± 1.15^b^	80.73 ± 2.21^c^	136.00 ± 1.41^b^	191.50 ± 2.12^b^
MwKP	8.40 ± 0.14^a^	6.85 ± 0.21^a^	62.09 ± 0. 30^a^	90.16 ± 0.23^b^	144.5 ± 0. 71^a^	198.50 ± 0.71^a^

**Flatbreads**	**Fe**	**Zn**	**Na**	**K**	**Ca**	**Mg**
T₀	3.14 ± 0.02^e^	3.20 ± 0.03^c^	2.95 ± 0.06^e^	378.75 ± 0.35^d^	30.25 ± 0.06^e^	124.65 ± 0.92^e^
T₁	3.36 ± 0.01^d^	3.39 ± 0.02^bc^	4.55 ± 0.07^d^	381.70 ± 0.99^c^	33.88 ± 0.03^d^	130.31 ± 0.98^d^
T₂	3.58 ± 0.03^c^	3.56 ± 0.03^b^	6.13 ± 0.10^c^	384.13 ± 1.23^bc^	37.84 ± 0.51^c^	135.29 ± 1.01^c^
T₃	3.78 ± 0.01^b^	3.81 ± 0.13^a^	7.75 ± 0.21^b^	386.26 ± 1.05^ab^	42.04 ± 1.35^b^	140.77 ± 1.74^b^
T₄	4.04 ± 0.08^a^	3.94 ± 0.08^a^	9.58 ± 0.59^a^	388.38 ± 0.87^a^	45.35 ± 0.92^a^	145.25 ± 1.06^a^

Values are expressed as means ± S.D. (*n* = 2). Mean values presenting similar lettering in a column are statistically non-significant at *p* < 0.05. T_0_ = Control, T_1_ = 2.5 % MwKP, T_2_ = 5 % MwKP, T_3_ = 7.5 % MwKP, T_4_ = 10 % MwKP flatbreads.

### 3.3 Antinutrients contents of RWF, RKP, MwKP and MwKP supplemented flatbreads

The study identified the RKP to uphold the highest levels of intrinsic toxicants including phytates, alkaloids, tannins and saponins i.e., 892, 1154, 926, and 549 mg/100 g. However, MwKP significantly (*p* < 0.05) reduced the load of these toxicants (Table 3). The overall rate of reduction for phytates, alkaloids, tannins and saponins was noted between 83 and 90 %. Similarly, the decline in loads of these antinutrients were also recorded individually; phytates from 891 to 115 mg/100 g (87 % reduction), alkaloids from 1154 to 196 mg/100 g (83 % reduction), tannins from 926 to 92 mg/100 g (90 % reduction) and saponins from 549 to 88 mg/100 g (84 % reduction). Unlike kachnar powder, the listed toxicants were not detected in RWF. Our results for the antinutrients of MwKP are in close corroboration with earlier researchers (Vijayakumari et al., 2007), wherein the study stated significant (*p* < 0.05) decline in tannins and phytates of *B. variegata* seed powder on treating with the soaking, cooking and hydrothermal methods. Comparable findings were observed by earlier researchers consisting of (Waseem et al., 2024) wherein the study reported the positive co-relation of microwave heat processing on reduction of antinutrients in spinach powder and reduced the phytates, tannins, and alkaloids by 88, 85 and 88 %, respectively.

**Table 3:** Antinutrient contents of RWF, RKP, MwKP and MwKP supplemented flatbreads (mg/100 g).

**Powders**	**Phytates**	**Alkaloids**	**Tannins**	**Saponins**
RWF	Nil	Nil	Nil	Nil
RKP	891.66 ± 7.42^a^	1154.00 ± 8.49^a^	926.47 ± 6.63^a^	549.39 ± 5.42^a^
MwKP	115.62 ± 4.58^b^	196.18 ± 2.46^b^	92.64 ± 5.97^b^	88.08 ± 2.15^b^
% reduction	*86*	*83*	*90*	*84*

**Flatbreads**	**Phytates**	**Alkaloids**	**Tannins**	**Saponins**
T₀	Nil	Nil	Nil	Nil
T₁	2.83 ± 0.09^d^	4.89 ± 0.02^d^	2.27 ± 0.08^d^	2.17 ± 0.04^d^
T₂	5.76 ± 0.04^c^	9.80 ± 0.01^c^	4.61 ± 0.04^c^	4.36 ± 0.06^c^
T₃	8.65 ± 0.04^b^	14.70 ± 0.01^b^	6.92 ± 0.04^b^	6.58 ± 0.04^b^
T₄	11.52 ± 0.06^a^	19.59 ± 0.04^a^	9.23 ± 0.05^a^	8.78 ± 0.05^a^

Values are expressed as means ± S.D. (*n* = 2). Mean values presenting similar lettering in a column are statistically non-significant at *p* < 0.05. T_0_ = Control, T_1_ = 2.5 % MwKP, T_2_ = 5 % MwKP, T_3_ = 7.5 % MwKP, T_4_ = 10 % MwKP flatbreads.

The antinutrients like phytates, oxalates, tannins, lectins and saponins are known to pose adverse health effects in humans such as hyperactivity, paralysis, kidney stones, hemolysis, antienzyme activities and even death (Eleazu et al., 2020). Phytates hinders protein and zinc, iron, calcium and magnesium absorption through chelation (Kataria et al., 2022). The data on MwKP supplemented flatbreads elucidated presence of very meagre concentrations of phytates, alkaloids, tannins and saponins i.e., 11.5, 20, 9.2 and 8.8 mg/100 g, respectively at maximum 10 % supplementation level. While, the least amount of the listed antinutrients were identified in flatbreads prepared by incorporating 2.5 % MwKP i.e., 2.8, 4.9, 2.2, and 2 mg/100 g, respectively. However, the antinutrients in MwKP supplemented flatbreads were under permissible safer limits i.e., phytates (25 mg/100 g), tannins (150 mg/100 g) and oxalates (100 mg/100 g) which allows the consumers to prefer the value-added flatbreads (Veer et al., 2021; Waseem et al., 2022a, Waseem et al., 2022b). The reduction of these antinutrients in plant food materials on microwave heating could be linked with the higher thermal degradations and weaker bonding with forces weaker hydrogen bonds (Waseem et al., 2024). An earlier study by (Waseem et al., 2022a) reported microwave heat treatment of potato powder resulted in decline in a load of alkaloids, oxalates, tannins and phytates as 14.5, 6.2, 14.6 and 7.9 mg/100 g, respectively. Likewise in another study by (Waseem et al., 2024) researchers found that among thermal and non-thermal treatment of spinach powder, the flatbreads prepared with the supplementation of microwave heated spinach powder showed highest % reduction in alkaloids, oxalates, tannins and phytates as 85, 87, 88 and 89 %, respectively.

### 3.4 Antioxidant activities in RFW, RKP, MwKP and MwKP supplemented flatbreads

The highest concentrations of DPPH, ABTS, FRAP, TPC, and TFC in RKP i.e., 107.8 μmol TE/g, 273 μmol TE/g, 23.4 mmol Fe^2+/^100 g, 47.8 mg GAE/g, and 6.5 mg CAE/g, respectively while the lowest contents of these antioxidants were observed in RWF i.e., 4.5 μmol TE/g, 2.5 umol/ TE/g, 2.4 mmol Fe^2+/^100 g, 1.7 mg GAE/g, and 0.7 mg CAE/g, respectively. However, the MwKP showed slightly lower mean values of DPPH, ABTS, FRAP, TPC, and TFC i.e., 100 μmol TE/g, 259 μmol TE/g, 21 mmol Fe^2+^/100 g, 42.4 mg GAE/g, and 5.9 mg CAE/g, respectively then RKP (Table 4). The presence of slightly lower antioxidants in MwKP samples could be attributed to the thermal degradation and Maillard reaction. However, our results are in line with the earlier research by (Kataria et al., 2022), wherein mean values for DPPH, ABTS, and FRAP of teff grains were observed to be reduced marginally from 27.5 to 25.2, 2.7–1.9, and 10.3–8.8 μmol TE/g, respectively. In another study the researchers have also reported that TPC and TFC of *S. hispanica* were also non-significantly reduced from 6.05 to 6.00 mg GAE/g and 50.27–46.65 mg QE/g, respectively on microwave treatment (Kataria et al., 2022; Sharma et al., 2022).

**Table 4:** Antioxidants of RWF, RKP, MwKP and MwKP supplemented flatbreads.

**Powders**	**DPPH** **(umol TE/g)**	**ABTS** **(umol TE/g)**	**FRAP** **(mmol Fe**^**2+**^**/100** **g)**	**TPC** **(mg GAE/g)**	**TFC** **(mg CAE/g)**
RWF	4.54 ± 0.11^b^	2.43 ± 0.06^b^	2.42 ± 0.16^b^	1.67 ± 0.11 b	0.73 ± 0.03^c^
RKP	107.84 ± 3.85^a^	273.00 ± 7.38^a^	23.41 ± 1.30^a^	47.76 ± 3.93^a^	6.58 ± 0.25^a^
MwKP	100.50 ± 1.32^a^	258.87 ± 4.38^a^	20.95 ± 1.37^a^	42.40 ± 1.50^a^	5.90 ± 0.11^b^

**Flatbreads**	**DPPH** **(umol TE/g)**	**ABTS** **(umol TE/g)**	**FRAP** **(mmol Fe**^**2+**^**/100** **g)**	**TPC** **(mg GAE/g)**	**TFC** **(mg CAE/g)**
T₀	3.79 ± 0.01^e^	2.16 ± 0.02^e^	2.29 ± 0.02^e^	1.14 ± 0.02^e^	0.65 ± 0.02^c^
T₁	6.27 ± 0.04^d^	8.39 ± 0.34^d^	2.75 ± 0.08^d^	2.19 ± 0.02^d^	0.77 ± 0.05^c^
T₂	8.62 ± 0.29^c^	14.85 ± 0.36^c^	3.23 ± 0.16^c^	3.16 ± 0.15^c^	0.93 ± 0.03^b^
T₃	11.07 ± 0.37^b^	20.88 ± 0.99^b^	3.67 ± 0.27^b^	4.22 ± 0.15^b^	1.04 ± 0.08^b^
T₄	13.41 ± 0.62^a^	27.45 ± 0.86^a^	4.28 ± 0.16^a^	5.22 ± 0.23^a^	1.21 ± 0.04^a^

Values are expressed as means ± S.D. (*n* = 2). Mean values presenting similar lettering in a column are statistically non-significant at *p* < 0.05. T_0_ = Control, T_1_ = 2.5 % MwKP, T_2_ = 5 % MwKP, T_3_ = 7.5 % MwKP, T_4_ = 10 % MwKP flatbreads.

The results for the MwKP supplemented flatbreads showed significant (*p* < 0.05) improvement in DPPH, ABTS, FRAP, TPC and TFC from 3.8 to 13.4 μmol TE/g, 2.2–27.4 μmol TE/g, 2.3–4.3 mmol Fe^2+^/100 g, 1.1–5.2 mg GAE/g, and 0.6–1.2 mg CAE/g, respectively at 0–10 % (T_0_-T_4_) supplementation levels (Table 4). The notable increase in antioxidants suggestive of the presence of higher magnitudes of antioxidants in MwKP itself. Our findings are in close agreement with previous study by (Ramashia et al., 2021), wherein the incorporation of *P. curatellifolia* peel powder increased the DPPH, FRAP, TPC and TFC in biscuits ranging from 48.7 to 94.7 %, 108–162 mg GAE/g, 20–48.5 mg GAE/g, and 0.028–0.104 mg CE/g, respectively. Higher antioxidant potential of kachnar and its supplemented baked goods could be linked with the presence of elevated levels of polyphenolics and flavonoids which are positively correlated with levels of improved antioxidant and free radical scavenging activities. In an earlier study by (Vijayan et al., 2019) elucidated promising health potentials of antioxidants which are linked to reduce cell damage and helpful in prevention of oxidative stress, malignant formation against cancer, and coronary heart diseases. In an earlier study by (Aljahani, 2022) it was revealed that the successive addition of yellow pumpkin flour in flatbreads at 5–15 % levels resulted in significant increase in total phenolics and DPPH values from 5.2 to 5.4 mg GAE/g and 5.8–6.4 %, respectively. Likewise, a study by (Tehreem et al., 2024) reported the increase of TPC and DPPH mean values from 27 to 35 mg GAE/100 g and 214–239 μmol T.E./100 g, respectively on replacement of 5 % wheat flour with pomegranate peel powder in flatbreads. The study also showed the highest DPPH and phenolics i.e., 285 μmol T.E./100 g and 38 mg GAE/100 g, respectively in flatbreads prepared with orange peel powder at 5 % replacement levels. However, another research by (Salve & Arya, 2020) elucidated, at 10 % replacement of wheat flour with peanut flour in flatbreads resulted in increase of TPC and TFC from 1.7 to 2.3 and 1.2–1.4 mg/g, respectively. Additionally, DPPH improved slightly from 0.81 to 0.82 mg/g and ABTS from 0.62 to 0.63 mg/g.

### 3.5 Carotenoids, tocopherols and textural measurements of RWF, RKP, MwKP and MwKP supplemented flatbreads

The results for total carotenoids and tocopherol contents among the RWF, RKP and MwKP elucidated the presence of the highest mean values in RKP i.e., 145 and 10.5 μg/g, respectively (Table 5). Our findings were in close agreement with the work of (Thakur et al., 2020), where in the researchers reported the values of total carotenoids and tocopherol contents in kachnar i.e., 142 and 10 μg/g, respectively. The results for the carotenoids and tocopherols of MwKP supplemented flatbreads portrayed significant (*p* *<* 0.05) increase from 1.3 to 14.1 and 4.2–5.04 μg/g for T_0_-T_4_, respectively. An earlier study by (Kaur et al., 2022) reported notable increase in carotenoid contents of flatbreads ranging from 40 to 160 μg/g on increasing the supplementation levels of red bell pepper powder in refined wheat flour. Likewise, another study by (Burešová et al., 2021) reported that the variation in carotenoid and tocopherol contents could be attributed to the higher thermostability characteristics.

**Table 5:** Carotenoids, tocopherols and textural measurements of RWF, RKP, MwKP and MwKP supplemented flatbreads.

**Powders**	**Carotenoids (μg/g)**	**Tocopherol (**μg**/g)**	**Hardness (N)**	**Gumminess (N, mm)**	**Springiness (mm)**	**Puffing (cm)**
RWF	2.70 ± 0.14^c^	8.85 ± 0.07^b^	–	–	–	–
RKP	145.30 ± 0.42^a^	10.50 ± 0.14^a^	–	–	–	–
MwKP	128.50 ± 2.12^b^	8.26 ± 0.06^c^	–	–	–	–

**Flatbreads**	**Carotenoids (μg/g)**	**Tocopherol (μg/g)**	**Hardness (N)**	**Gumminess (N, mm)**	**Springiness (mm)**	**Puffing (cm)**
T₀	1.35 ± 0.07^e^	4.25 ± 0.35^c^	4.15 ± 0.07^e^	3.095 ± 0.15^a^	1.3 ± 0.03^a^	5.05 ± 0.07^a^
T₁	4.78 ± 0.31^d^	4.48 ± 0.03^bc^	5.2 ± 0.14^d^	2.69 ± 0.13^b^	1.05 ± 0.07^b^	4.73 ± 0.04^b^
T₂	7.89 ± 0.16^c^	4.63 ± 0.04^abc^	6.46 ± 0.34^c^	2.05 ± 0.07^c^	0.915 ± 0.01^c^	4.15 ± 0.07^c^
T₃	11.09 ± 0.15^b^	4.83 ± 0.05^ab^	7.55 ± 0.49^b^	1.625 ± 0.04^d^	0.745 ± 0.02^d^	3.73 ± 0.04^d^
T₄	14.10 ± 0.14^a^	5.04 ± 0.05^a^	8.78 ± 0.31^a^	1.415 ± 0.02^d^	0.585 ± 0.02^e^	3.11 ± 0.01^e^

Values are expressed as means ± S.D. (*n* = 2). Mean values presenting similar lettering in a column are statistically non-significant at *p* < 0.05. T_0_ = control, T_1_ = 2.5 % MwKP, T_2_ = 5 % MwKP, T_3_ = 7.5 % MwKP, T_4_ = 10 % MwKP flatbreads.

The results for the textural qualities of the MwKP supplemented flatbreads reported noticeable decline in gumminess and springiness from 3.1 to 1.4 (N, mm), 1.3–0.6 mm, respectively on increasing the supplementation levels from 0 to 10 % (i.e., T_0_-T_4_). However, the results for the hardness of the supplemented flatbreads portrayed significant (*p* < 0.05) increase from 4.15 to 8.78 N on enhancing the supplementation levels from 0 to 10 % (i.e., T_0_-T_4_).The similar trend was also observed in an earlier study by (Koksel et al., 2024) wherein, the researchers reported significant (*p* < 0.05) increase in hardness from 0.4 to 3.2 N at 6 % substitution with barley and observed notable decline in the springiness (0.93–0.87 mm) of the barley-bazlama flour composite flatbreads at same substitution level.

Puffing is a viable parameter of quality which indicates physical quality of baked goods. A significant (*p* < 0.05) decrease in puffing height of the MwKP flatbreads was observed from 5 to 3 cm at 0–10 % supplementation levels (i.e., T_0_-T_4_). An earlier study by (Waseem et al., 2022b), elucidated similar decline in puffing height of flatbreads from 6.4 to 2.7 cm on addition of potato powder from 0 to 10 %. However, the reduction in puffing heights of the supplemented flatbreads could be linked with the presence of higher magnitudes of dietary fibers, non-gluten proteins, higher intermolecular forces, and poor water-holding ability of non-wheat flours resulting in a low steam formation which subsequently linked with the reduced puffed height of flatbreads (Waseem et al., 2022b).

### 3.6 Sensory evaluation of RWF and MwKP supplemented flatbreads

**Fig. 1:**
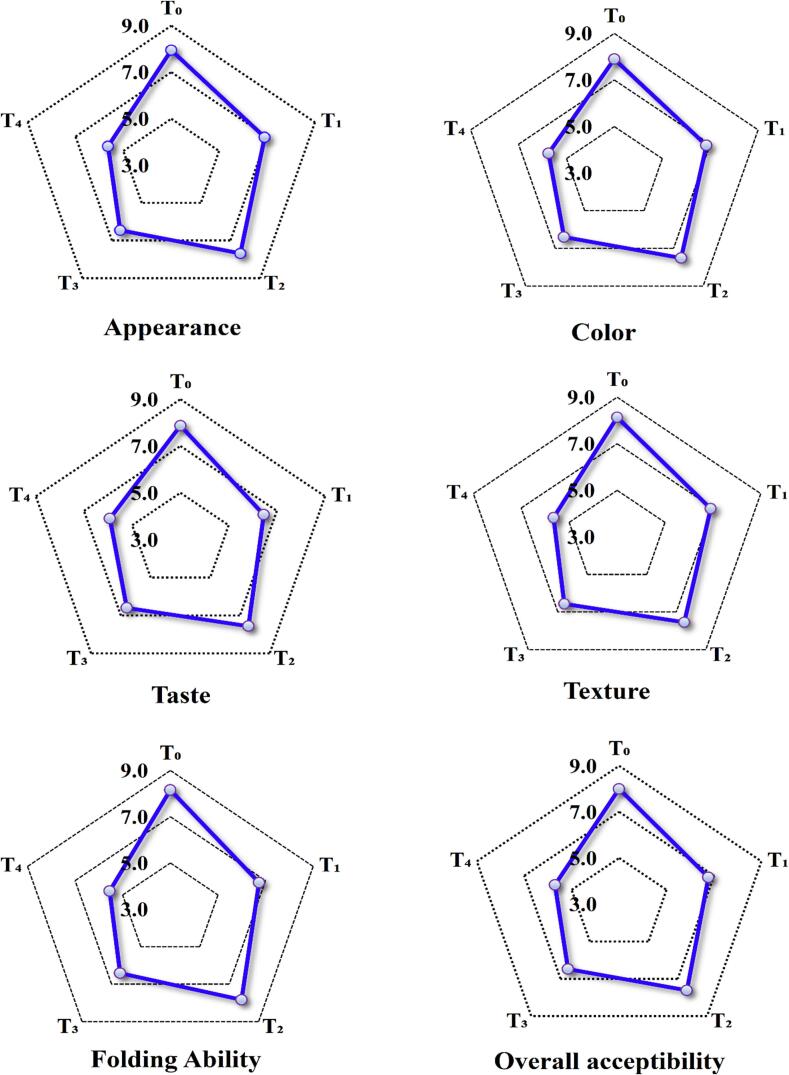
Sensory evaluation of refined wheat flour (RWF) and microwave processed kachnar powder (MwKP) supplemented flatbreads. T_0_ = 100 % RWF flatbreads (Control). T_1_ = 2.5 % MwKP. T_2_ = 5 % MwKP. T_3_ = 7.5 % MwKP. T_4_ = 10 % MwKP flatbreads.

Value addition of natural ingredients in development of baked goods are often anticipated to have a profound impact on sensory characteristics and consumer acceptability. The results for the sensorial parameters of the flatbreads elucidated the highest sensory scores for color, taste, texture and overall acceptability for control i.e., 7.9, 7.8, 8.1 and 8.1, respectively. However, among all the treatments, T_3_ exhibited the highest sensory scores for color, taste, texture and overall acceptability i.e., 7.5, 7.6, 7.5 and 7.6, respectively (Fig. 1). The sensory experts preferred T_2_ (i.e., 5 % supplementation level) for all sensory parameters as it reported the highest sensory scores. However, the results for sensory scores of flatbreads revealed significant decline in sensory scores of all sensory parameters on enhancing the MwKP supplementation ≥7.5 %; as it portrayed astringent and undesirable sensory flavor when compared to the control. The bitter taste in the flatbreads at ≥7.5 % could be due to the presence of higher amounts of tannins and ellagitannins. An earlier study by (Gupta et al., 2017) also reported comparable findings for sensory characteristics i.e., color, taste and texture of *Uttapam* (i.e., an Indian food product)prepared with supplementing the kachnar leaves powder. Likewise, another study conducted by (Khan et al., 2023) also elucidated significant (*p* < 0.05) improvement in sensory parameters however, the study also indicated notable decline in overall acceptability from 7.8 to 6.1 in *moringa oleifera* powder-based flatbreads prepared at 0–10 % supplementation levels. A study by (El-Sohaimy et al., 2019) was found to affect the sensory scores of flavor, texture and taste of flatbreads on increasing the supplementation levels beyond 10 %.

## 4 Conclusions

Supplementation of MwKP in flatbreads resulted in improved nutritional profile i.e., ash, fiber, protein, Fe, Zn, Na, K, and Ca of baked good which thereby indicating the commercial applicability of MwKP as a novel ingredient of choice in development of value-added baked products. The key results also indicated a notable increase in antioxidant activities of MwKP and supplemented flatbreads. Sensory experts preferred the flatbreads prepared with 5 % MwKP supplementation owing to better sensory appeals for color, taste and texture. In the conclusion, present study strongly recommends the use of microwave heat processing, particularly at 0.8 kW for 3 min as an effective technique in mitigating the intrinsic toxicants i.e., antinutrients. Findings of present study emphasize the potential use of MwKP as a viable and safer ingredient for use in preparation of gravies, soups, salads and ready to eat foods. MwKP as an ingredient holds promising scope in addressing micronutrients deficiencies and may help in relieving several health disorders such as cancer, diabetes and obesity.

## Ethical statement

All the sensory experts available at the Department of Food Science and Technology were taken on board before sensory evaluation of baked flatbreads samples. Privacy and rights of all the sensory experts were kept in secret during entire sensory evaluation process. All flatbreads' samples used in the sensory evaluation study were safe for human consumption. The sensory experts committee was approved by the Chairman, Department of Food Science and Technology.

## CRediT authorship contribution statement

**Syed Hammad Mazhar:** Writing – review & editing, Writing – original draft, Software, Project administration, Formal analysis, Conceptualization. **Zulfiqar Ahmad:** Writing – review & editing, Writing – original draft, Software, Project administration, Formal analysis, Data curation, Conceptualization. **Muhammad Rizwan Javed:** Writing – review & editing, Writing – original draft, Validation, Software, Formal analysis, Data curation, Conceptualization. **Muhammad Ammar Khan:** Writing – review & editing, Writing – original draft, Resources, Methodology, Conceptualization. **Robert Mugabi:** Writing – review & editing, Resources, Investigation, Formal analysis, Conceptualization. **Tawfiq Alsulami:** Writing – review & editing, Visualization, Software, Resources, Project administration, Funding acquisition. **Gulzar Ahmad Nayik:** Writing – review & editing, Writing – original draft, Supervision, Resources, Investigation, Formal analysis, Data curation.

## Declaration of competing interest

The authors declare that they have no known competing financial interests or personal relationships that could have appeared to influence the work reported in this paper.

## Data Availability

All the authors declare that if more data is required, then the data will be provided on a request basis.

## References

[bb0005] Ahmed S., Ahmad U., Hameed S., Azher S., Malik A. (2023). Exploring the anti-anemic potential of Bauhinia variegata Linn leaves powder biscuits in animal modeling: Anti-anemic potential of Bauhinia variegate Linn. DIET FACTOR (Journal of Nutritional and Food Sciences).

[bb0010] Ali S.M., Usman A., Wazir M.A., Shaheer T., Manzoor R., Abbas K. (2021). Pharmacognostic, phytochemical and biological investigations of Bauhinia variegata (L). Fruit. *Hamdard*. Medicus.

[bb0015] Aljahani A.H. (2022). Wheat-yellow pumpkin composite flour: Physico-functional, rheological, antioxidant potential and quality properties of pan and flat bread. Saudi Journal of Biological Sciences.

[bb0020] Awasthi M., Verma R. (2019). Exploration of *Bauhinia variegata* (kachnar) and *Cordia dichotoma* (lesora) for their mineral content. Asian Journal of Dairy and Food Research.

[bb0025] Bhandari J., Thapa P., Niraula P., Thapa N., Shrestha N., Shrestha B.G. (2017). Study of phytochemical, anti-microbial, anti-oxidant, phytotoxic, and immunomodulatory activity properties of Bauhinia variegata. Journal of Tropical Life Science.

[bb0030] Bhavya S.N., Prakash J. (2021). Nutritional properties of iron fortified flatbreads enriched with greens and legumes. Journal of Food Processing and Preservation.

[bb0035] Bratovcic A., Djapo-Lavic M., Kazazic M., Mehic E. (2021). Evaluation of antioxidant capacities of orange, lemon, apple and banana peel extracts by frap and abts methods. Roumanian Journal of Chemistry.

[bb0040] Burešová B., Paznocht L., Kotíková Z., Giampaglia B., Martinek P., Lachman J. (2021). Changes in carotenoids and tocols of colored-grain wheat during unleavened bread preparation. Journal of Food Composition and Analysis.

[bb0045] Eleazu C.O., Eleazu K.F., Ukamaka G., Adeolu T., Ezeorah V., Ezeorah B., Ilom J. (2020). Nutrient and antinutrient composition and heavy metal and phenolic profiles of maize (Zea mays) as affected by different processing techniques. ACS Food Science & Technology.

[bb0050] Elfeky W., Mohamed E., Hussien H. (2024). Nutritional properties of high protein pancakes using Sorghum and sweet lupine. Food Technology Research Journal.

[bb0055] El-Sohaimy S., Shehata M., Mehany T., Zeitoun M. (2019). Nutritional, physicochemical, and sensorial evaluation of flat bread supplemented with quinoa flour. International Journal of Food Science.

[bb0060] Faizal F., Ahmad N., Yaacob J., Halim-Lim S.A., Rahim M.A. (2023). Food processing to reduce antinutrients in plant-based foods. International Food Research Journal.

[bb0065] Gupta A., Tripathi J., Yadav N., Sagar P. (2017). Ingredient substitution and quality enrichment of conventional Indian recipes with underutilized fresh Kachnar leaves. http://nopr.niscpr.res.in/handle/123456789/42855.

[bb0070] Hussnain A., Ahmad S., Murtaza S., Iqbal S., Farooq U., Sibt-e-Abbas M. (2022). Development of quinoa and sorghum supplemented flatbread. Journal of Food Processing and Preservation.

[bb0075] Ifeanacho M., Ezecheta C. (2020). Effect of domestic food processing methods on anti nutrients, some mineral content and functional properties of mungbean (*Vigna radiata*) flours. Journal of Dieticians Association of Nigeria.

[bb0080] Jr L. (2019).

[bb0085] Kamal Y., Khan T., Haq I., Zahra S., Asim M., Shahzadi I., Mannan A., Fatima N. (2022). Phytochemical and biological attributes of *Bauhinia variegata* L. (Caesalpiniaceae). Brazilian Journal of Biology.

[bb0090] Kansal M., Shukla P., Shukla P. (2020). A boon to human health- *Bauhinia variegata*. International Journal of Pharmacognosy.

[bb0095] Kataria A., Sharma S., Singh A., Singh B. (2022). Effect of hydrothermal and thermal processing on the antioxidative, antinutritional and functional characteristics of *Salvia hispanica*. Journal of Food Measurement and Characterization.

[bb0100] Kaur A., Aggarwal P., Kaur N., Kaur S. (2022). Enhancement of biofunctional properties, sensory attributes, and shelf life of flatbread by incorporating red bell pepper (Capsicum annuum L.). Journal of Food Processing and Preservation.

[bb0105] Khan M.A., Shakoor S., Ameer K., Farooqi M.A., Rohi M., Saeed M., Tanweer S. (2023). Effects of dehydrated moringa (*moringa oleifera*) leaf powder supplementation on physicochemical, antioxidant, mineral, and sensory properties of whole wheat flour leavened bread. Journal of Food Quality.

[bb0110] Khare P., Kishore K., Sharma D.K. (2018). Historical aspects, medicinal uses, phytochemistry and pharmacological review of Bauhinia variegata. Asian Journal of Pharmacy and Pharmacology.

[bb0115] Kiumarsi M., Shahbazi M., Yeganehzad S., Majchrzak D., Lieleg O., Winkeljann B. (2019). Relation between structural, mechanical and sensory properties of gluten-free bread as affected by modified dietary fibers. Food Chemistry.

[bb0120] Koksel H., Tekin-Cakmak Z.H., Oruc S., Kilic G., Ozkan K., Cetiner B., Jilal A. (2024). A new functional wheat flour flatbread (Bazlama) enriched with high-β-glucan hull-less barley flour. Foods.

[bb0125] López-Moreno M., Garcés-Rimón M., Miguel M. (2022). Antinutrients: Lectins, goitrogens, phytates and oxalates, friends or foe?. Journal of Functional Foods.

[bb0130] Park J., Do S., Lee M., Ha S., Lee K.-G. (2022). Preparation of turmeric powder with various extraction and drying methods. Chemical and Biological Technologies in Agriculture.

[bb0135] Ramashia S.E., Mamadisa F.M., Mashau M.E. (2021). Effect of Parinari curatellifolia peel flour on the nutritional, physical and antioxidant properties of biscuits. Processes.

[bb0140] Salve A.R., Arya S. (2020). Bioactive constituents, microstructural and nutritional quality characterisation of peanut flat bread. Journal of Food Measurement and Characterization.

[bb0145] Seed K., Balogun B. (2013). Effects of processing methods on anti-nutrient levels of *Bauhinia monandra*. Polymers for Advanced Technologies.

[bb0150] Sharma K., Kumar V., Kumar S., Pinakin D.J., Babbar N., Kaur J., Sharma B.R. (2022). Process optimization for drying of Bauhinia variegata flowers: Effect of different pre-treatments on quality attributes. Journal of Food Processing and Preservation.

[bb0155] Sharma K., Kumar V., Kumar S., Sharma R., Mehta C. (2021). *Bauhinia variegata*: A comprehensive review on bioactive compounds, health benefits and utilization. Advances in Traditional Medicine.

[bb0160] Stoleru V., Jacobsen S.-E., Vitanescu M., Jitareanu G., Butnariu M., Munteanu N., Mihalache G. (2022). Nutritional and antinutritional compounds in leaves of quinoa. Food Bioscience.

[bb0165] Suhag R., Dhiman A., Deswal G., Thakur D., Sharanagat V.S., Kumar K., Kumar V. (2021). Microwave processing: A way to reduce the anti-nutritional factors (ANFs) in food grains. Lebensmittel-Wissenschaft & Technologie.

[bb0170] Tehreem S., Abbas M., Khan I., Nayab D., Shah M. (2024). Health benefits of wheat flatbreads with different fruit peels; determination of anti-oxidant, nutritional value and sensory characteristics. International Journal of Pathology.

[bb0175] Thakur A., Singh S., Puri S. (2020). Nutritional evaluation, phytochemicals, antioxidant and antibacterial activity of gerardiana diversifolia Linn. And bauhinia variegata Linn. Wild edible plants of western himalayas. Wild Edible Plant Western Himalayas.

[bb0180] Tripathi A.K., Gupta P.S., Singh S.K. (2019). Antidiabetic, anti-hyperlipidemic and antioxidant activities of Bauhinia variegata flower extract. Biocatalysis and Agricultural Biotechnology.

[bb0185] Veer S., Pawar V., Kambale R. (2021). Antinutritional factors in foods. The Pharma Innovation Journal.

[bb0190] Vijayakumari K., Pugalenthi M., Vadivel V. (2007). Effect of soaking and hydrothermal processing methods on the levels of antinutrients and in vitro protein digestibility of *Bauhinia purpurea* L. seeds. Food Chemistry.

[bb0195] Vijayan R., Joseph S., Mathew B. (2019). Anticancer, antimicrobial, antioxidant, and catalytic activities of green-synthesized silver and gold nanoparticles using *Bauhinia purpurea* leaf extract. Bioprocess and Biosystems Engineering.

[bb0200] Waseem M., Akhtar S., Ahmad N., Ismail T., Lazarte C.E., Hussain M., Manzoor M.F. (2022). Effect of microwave heat processing on nutritional indices, antinutrients, and sensory attributes of potato powder-supplemented flatbread. Journal of Food Quality.

[bb0205] Waseem M., Akhtar S., Ahmad N., Ismail T., Lazarte C.E., Hussain M., Manzoor M.F. (2022). Effect of microwave heat processing on nutritional indices, antinutrients, and sensory attributes of potato powder-supplemented flatbread. Journal of Food Quality.

[bb0210] Waseem M., Akhtar S., Manzoor M.F., Mirani A.A., Ali Z., Ismail T., Karrar E. (2021). Nutritional characterization and food value addition properties of dehydrated spinach powder. Food Science & Nutrition.

[bb0215] Waseem M., Akhtar S., Mehmood T., Qamar M., Saeed W., Younis M., Perveen S., Ismail T., Esatbeyoglu T. (2024). Nutritional, safety and sensory quality evaluation of unleavened flatbread supplemented with thermal and non-thermal processed spinach powder. Journal of Agriculture and Food Research.

[bb0220] Weremfo A., Adulley F., Dabie K., Abassah-Oppong S., Peprah-Yamoah E. (2022). Optimization of ultrasound-assisted extraction of phenolic antioxidants from Turkey berry (*Solanum torvum* Sw) fruits using response surface methodology. Journal of Applied Research on Medicinal and Aromatic Plants.

[bb0225] Wu Z., Meenu M., Xu B. (2021). Nutritional value and antioxidant activity of Chinese black truffle (*tuber indicum*) grown in different geographical regions in China. Lebensmittel-Wissenschaft & Technologie.

